# Cortical *N*‐acetylaspartate concentrations are impacted in chronic stroke but do not relate to motor impairment: A magnetic resonance spectroscopy study

**DOI:** 10.1002/hbm.25421

**Published:** 2021-05-03

**Authors:** Jennifer K. Ferris, Jason L. Neva, Irene M. Vavasour, Kaitlin J. Attard, Brian Greeley, Kathryn S. Hayward, Katie P. Wadden, Alex L. MacKay, Lara A. Boyd

**Affiliations:** ^1^ Department of Physical Therapy University of British Columbia Vancouver British Columbia Canada; ^2^ École de Kinésiologie et des Sciences de l'activité Physique Université of Montréal Montreal Quebec Canada; ^3^ Centre de Recherche de l'Institut Universitaire de Gériatrie de Montréal CIUSSS Centre‐sud‐de‐I'île de Montréal Montreal Quebec Canada; ^4^ Faculty of Medicine, UBC MRI Research Center University of British Columbia Vancouver BC Canada; ^5^ School of Health Sciences, Florey Institute of Neuroscience and Mental Health, NHMRC CRE in Stroke Rehabilitation and Brain Recovery The University of Melbourne Parkville Victoria Australia; ^6^ Faculty of Medicine Memorial University of Newfoundland St. John's Newfoundland Canada

**Keywords:** hemiparesis, magnetic resonance spectroscopy, motor cortex, movement, somatosensory cortex, stroke, upper extremity

## Abstract

Magnetic resonance spectroscopy (MRS) measures cerebral metabolite concentrations, which can inform our understanding of the neurobiological processes associated with stroke recovery. Here, we investigated whether metabolite concentrations in primary motor and somatosensory cortices (sensorimotor cortex) are impacted by stroke and relate to upper‐extremity motor impairment in 45 individuals with chronic stroke. Cerebral metabolite estimates were adjusted for cerebrospinal fluid and brain tissue composition in the MRS voxel. Upper‐extremity motor impairment was indexed with the Fugl‐Meyer (FM) scale. *N*‐acetylaspartate (NAA) concentration was reduced bilaterally in stroke participants with right hemisphere lesions (*n* = 23), relative to right‐handed healthy older adults (*n* = 15; *p* = .006). Within the entire stroke sample (*n* = 45) NAA and glutamate/glutamine (GLX) were lower in the ipsilesional sensorimotor cortex, relative to the contralesional cortex (NAA: *p* < .001; GLX: *p* = .003). Lower ipsilesional NAA was related to greater extent of corticospinal tract (CST) injury, quantified by a weighted CST lesion load (*p* = .006). Cortical NAA and GLX concentrations did not relate to the severity of chronic upper‐extremity impairment (*p* > .05), including after a sensitivity analysis imputing missing metabolite data for individuals with large cortical lesions (*n* = 5). Our results suggest that NAA, a marker of neuronal integrity, is sensitive to stroke‐related cortical damage and may provide mechanistic insights into cellular processes of cortical adaptation to stroke. However, cortical MRS metabolites may have limited clinical utility as prospective biomarkers of upper‐extremity outcomes in chronic stroke.

## 1 INTRODUCTION

Upper‐extremity motor impairments are a significant source of chronic disability after stroke (Veerbeek, Kwakkel, van Wegen, Ket, & Heymans, 2011). The most typical cause of upper‐extremity impairment is damage to the corticospinal tract (CST; Feng et al., 2015; Lindenberg et al., 2010). CST damage induces structural and functional changes in ipsilesional as well as contralesional sensorimotor cortices. Notably, there is a loss of tissue integrity that extends into the spared ipsilesional cortex (Cheng et al., 2015) and increased functional activity in the contralesional cortex (Schaechter & Perdue, 2008). Our understanding of cortical changes after stroke relies primarily on evidence from neuroimaging methods such as structural volumetrics, diffusion tensor imaging, or functional magnetic resonance imaging (fMRI; for review see: Auriat, Neva, Peters, Ferris, & Boyd, 2015). These imaging methods are chemically nonspecific, meaning we must infer what results indicate for cortical neurobiology or metabolism after stroke. Magnetic resonance spectroscopy (MRS) is therefore an attractive technique to investigate cortical changes poststroke, as it quantifies the concentration of cerebral metabolites in vivo. MRS can directly assess the neurometabolic correlates of changes in cortical structure or function, which stands to increase our understanding of the neurobiological consequences of injury to the CST and processes underpinning stroke recovery.

Previous MRS studies in individuals with chronic stroke (>6 months poststroke) measured cerebral metabolite concentrations from primary and secondary sensorimotor cortices, which contribute to upper‐extremity sensorimotor function (Carlson, MacMaster, Harris, & Kirton, 2017; Cirstea et al., 2011; Craciunas et al., 2013; Jones, Borich, Vavasour, Mackay, & Boyd, 2016). Findings from these studies have generally been congruent with hypothesized patterns of cortical neurochemical changes in chronic stroke, based on inference from other neuroimaging and noninvasive brain stimulation modalities (Auriat et al., 2015). *N*‐acetylaspartate (NAA), a marker of intact neurons, is lower in the ipsilesional relative to contralesional cortex (Carlson et al., 2017; Cirstea et al., 2011; Craciunas et al., 2013; Jones et al., 2016), and its concentration in ipsi‐ and contralesional cortices relates to upper‐extremity motor outcomes (Carlson et al., 2017; Cirstea et al., 2011; Craciunas et al., 2013; Jones et al., 2016). Glutamate/glutamine (GLX), a principle excitatory neurotransmitter, is also lower in ipsilesional relative to contralesional cortex in the chronic phase of stroke (Cirstea et al., 2014; Jones et al., 2016). These findings reflect a pattern of neuronal loss in the ipsilesional hemisphere and increased neuronal excitation in the contralesional hemisphere. Furthermore, they provide preliminary evidence that sensorimotor NAA concentration may be a candidate biomarker of upper‐extremity outcome (Boyd et al., 2017).

MRS can measure neurochemical changes after stroke, however, there are important methodological challenges to MRS measurement in the cortex. MRS metabolite concentrations vary between tissue types (e.g., gray matter vs. white matter; Jansen, Backes, Nicolay, & Kooi, 2006), and cortical MRS voxels are expected to contain some proportion of cerebrospinal fluid (CSF). Our group previously reported ipsilesional NAA concentrations relate to the thickness of the motor cortex (Jones et al., 2016); while this supports the validity of NAA as a neuronal marker it also raises the possibility that hemispheric differences in cortical metabolite concentrations may reflect loss of ipsilesional cortical tissue (Brodtmann et al., 2012; Cheng et al., 2015) rather than true changes in metabolite concentrations. In the present analysis, we corrected cerebral metabolite estimates for the variations in water T1 and T2 of brain tissue types (gray matter, white matter and lesion) and CSF in the MRS voxel (Macmillan et al., 2016; Meyers et al., 2016). This is a more rigorous adjustment to MRS metabolite estimates than has been applied in previous MRS studies on cortical metabolites in chronic stroke, which have instead normalized metabolite concentrations by a ratio of total brain tissue in the voxel (Cirstea et al., 2011, 2014; Craciunas et al., 2013).

The current paper seeks to extend previous MRS research by examining metabolite concentrations from an upper‐extremity region of the primary motor and primary somatosensory cortex (hereafter referred to as “sensorimotor cortex”). We included a large sample of individuals with chronic stroke (*n* = 45) relative to previous MRS studies (the largest sample in past work was *n* = 20; Craciunas et al., 2013). Additionally, our sample included individuals with a range of stroke lesion locations and sizes (cortical, subcortical, and brainstem/cerebellum), providing a representative sample of individuals with chronic upper‐extremity motor impairment poststroke. We investigated how stroke impacts cortical metabolites by comparing concentrations between stroke participants and a cohort of healthy older adults, and between cerebral hemispheres within the stroke sample. Next, we investigated the relationship between CST lesion load and cortical metabolite concentrations. Finally, we tested whether cerebral metabolites relate to chronic upper‐extremity impairment and performed a sensitivity analysis to explore whether individuals with missing data due to large cortical lesions impacted observed relationships between cerebral metabolites and upper‐extremity impairment. We hypothesized that NAA concentrations would be reduced in the stroke group relative to healthy controls, and in the ipsilesional hemisphere relative to the contralesional hemisphere. Furthermore, we hypothesized that NAA concentrations would relate to extent of CST injury and degree of upper‐extremity impairment in our stroke cohort. We also explored changes to four other cerebral metabolites (GLX, myo‐inositol, creatine, and choline) without specific hypotheses about the direction of stroke‐related effects.

## 2 MATERIALS AND METHODS

### 2.1 Participants

We recruited individuals in the chronic phase of recovery (>6 months poststroke) after a first clinically diagnosed stroke, and a group of right‐hand dominant healthy adults. Participants were between the ages of 30–85. Participants were recruited from community postings as part of an intervention study conducted in the Brain Behavior Lab (Neva et al., 2019). MRS data and motor impairment scores were collected at the baseline timepoint for participants as a secondary research aim. Participants were excluded if they: (a) had a history of seizure/epilepsy, head trauma, a major psychiatric diagnosis, neurodegenerative disorders, or substance abuse; or (b) reported any contraindications to MRI. Informed consent was obtained for each participant in accordance with the Declaration of Helsinki. The Montreal Cognitive Assessment (MoCA) was administered to screen for cognitive impairment. If participants scored ≥24 on the MoCA study participants provided informed consent, if participants scored <24 on the MoCA their caregiver provided informed consent and the participant provided informed assent. The University of British Columbia research ethics board approved all aspects of the study protocol.

Motor impairment was assessed with the upper‐extremity portion of the Fugl‐Meyer (FM) scale (Fugl‐Meyer, Jääskö, Leyman, & Olsson, 1975; Sanford, Moreland, Swanson, Stratford, & Gowland, 1993). The FM is scored from 0 to 66 points with higher scores indicating less upper‐extremity impairment.

### 2.2 Magnetic resonance imaging acquisition

MRI scans were acquired at the University of British Columbia MRI Research Center on a Philips Achieva 3.0 T whole body MRI scanner (Philips Healthcare, Best, The Netherlands), with an eight‐channel sensitivity encoding head coil and parallel imaging. All participants received a three‐dimensional high‐resolution T_1_‐weighted MPRAGE anatomical scan (Shot *T*_R_ = 1950 ms, *T*_E_ = 3.65 ms, flip angle θ = 6°, SENSE factor = 2, FOV = 256 × 256 mm, 160 slices, 1 mm^3^ isotropic voxel).

### 2.3 Magnetic resonance spectroscopy (MRS)

**FIGURE 1:**
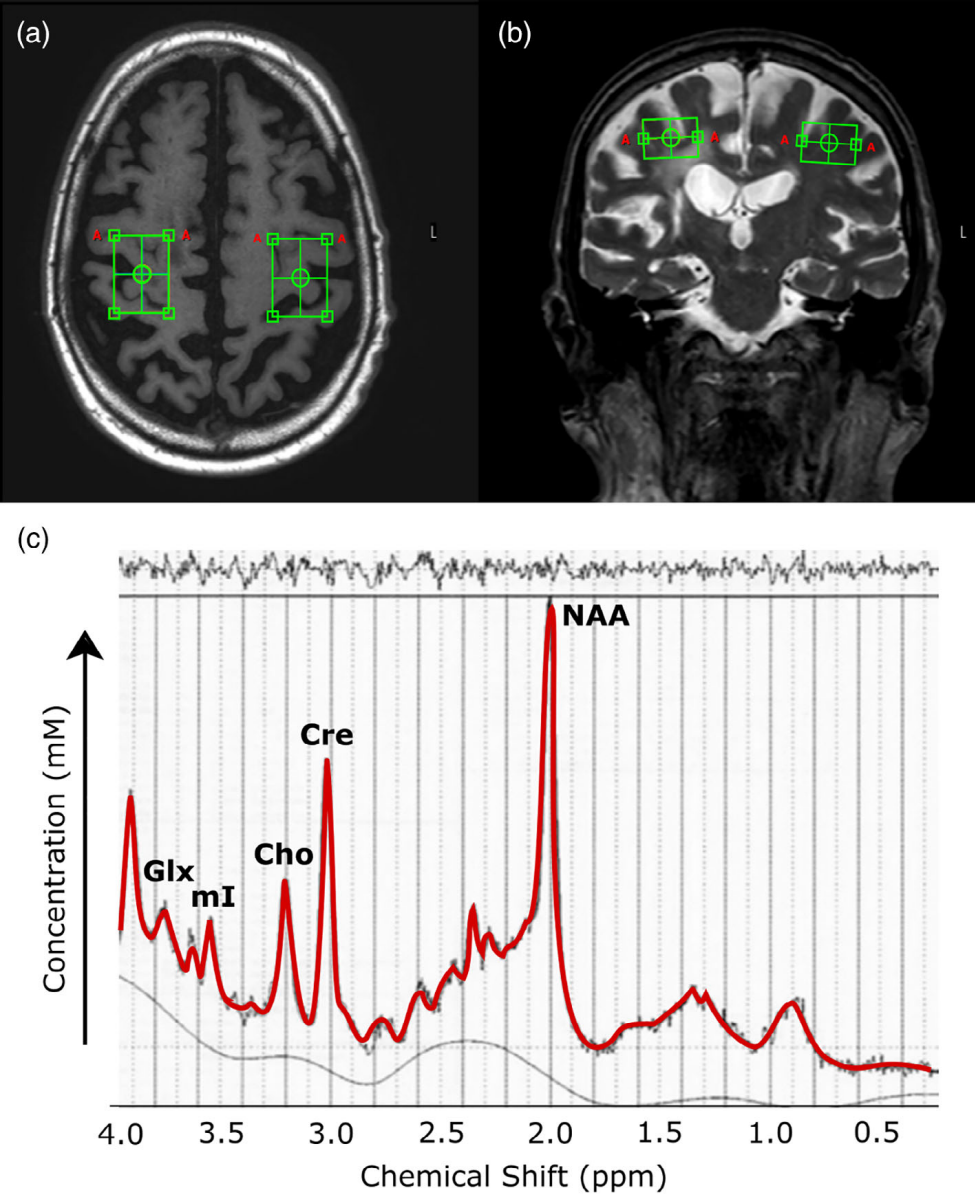
(a,b) MRS voxel placement for a participant with chronic stroke, over (a) T1 and (b) T2 scans. (c) Visualization of 1H‐MRS spectra for a representative participant. Cho, choline; Cre, creatine; GLX, glutamate/glutamine; mI, myo‐inositol; NAA, *N*‐acetylaspartate

A single‐voxel ^1^H‐MRS PRESS spectra was acquired (*T*_R_ = 2000 ms, *T*_E_ = 35 ms, sampling frequency = 2000 Hz, data points = 1024, signal averages = 128, voxel dimensions = 30 mm × 22 mm × 15 mm). The voxel was manually placed by trained researchers (by J.K.F. and K.P.W.) and was centered over the “hand knob” representation of the primary motor cortex (Yousry et al., 1997; Figure 1). Note that while the voxel was centered over the hand knob representation it included mixed gray and white matter from both the primary motor and primary somatosensory cortex (see Figure 1). For participants with stroke lesions that fell within the voxel of interest (i.e., strokes involving the sensorimotor cortex) we avoided including lesioned tissue in the voxel whenever possible with small adjustments to the voxel's rotation along the x or z plane without deviating from centering the voxel over the hand knob. For large cortical stroke lesions, where it was impossible to avoid placing the voxel over lesion, the MRS voxel was placed in the homologous region contralateral to the hand knob of the intact hemisphere. Automated second‐order projection‐based shimming was performed, and unsuppressed water spectra were acquired to enable metabolite concentration estimates. MRS files were processed using LCModel (Provencher, 2001), and water scaling was used to estimate absolute metabolite concentrations using the reference area of the unsuppressed water signal. The quality of spectral fits were first visually inspected to ensure goodness‐of‐fit and a narrow full width at half maximum (<0.05) and reasonable signal to noise (>20; by I.M.V.). Metabolite concentrations were rejected based on the Cramér‐Rao lower bound (CRLB) estimations from LCModel when CRLB estimations were >25% of the median value of the metabolite concentrations (Kreis, 2016). Absolute concentrations of MRS metabolites were determined by LCModel and used towards analysis (Provencher, 2001). At 3.0 T it is possible to reliably quantify the peaks of five cerebral metabolites with the ^1^H‐MRS PRESS spectra, and we included all five metabolites towards analysis: NAA, creatine, choline, myo‐inositol (mI), and the sum of the glutamate + glutamine peaks (GLX). Glutamate and glutamine have similar magnetic resonance spectra and thus their peaks were combined as GLX (Ramadan, Lin, & Stanwell, 2013).

FMRIB's FAST was used on T1 images to segment images into four tissue classes: gray matter, white matter, CSF, and dark non‐CSF tissue (i.e., lesion; Zhang, Brady, & Smith, 2001). MRS voxels were linearly registered into T1 space using FMRIB's FLIRT (Jenkinson, Bannister, Brady, & Smith, 2002) and used to define a region of interest from which FAST‐segmented tissue volumes were extracted. The accuracy of FAST segmentation in the MRS voxel was visually checked by a single rater (by K.J.A.) to ensure accuracy of tissue classification. Tissue volume fractions within the MRS voxels were used to adjust MRS metabolite concentration estimates for water relaxation using literature values (Macmillan et al., 2016; Meyers et al., 2016). The formulas for adjusting MRS metabolite signal based on the water content and T1/T2 relaxation values of tissue components are provided in the Data S1.

### 2.4 Corticospinal tract lesion load

Stroke lesions were manually drawn on the T1 scan by a single experienced researcher (by J.K.F.). T1 scans were nonlinearly registered to MNI152 1 mm space with FSL's FNIRT, and a binarized mask of the CST from the JHU white matter atlas (Hua et al., 2008) was generated with a threshold of 10. Overlap between the stroke lesion and the CST mask was calculated in MNI space. A weighted CST lesion load was computed according to previously published methods (Feng et al., 2015; Findlater et al., 2019). This metric accounts for the narrowing of the CST at the internal capsule by weighting the cross‐sectional area of the CST in the lesion load calculation.

### 2.5 Statistical analysis

Statistical analyses were conducted using R (v 4.0.2). Assumptions of normality were confirmed with Shapiro–Wilk tests. Unless otherwise indicated, *p* ≤ .05 was considered statistically significant. For stroke participants, we first evaluated the importance of adjusting metabolite concentration estimates for T1 and T2 relaxation values of tissue components by testing whether FAST segmentation volumes (CSF, gray matter, white matter, and lesion) in the MRS voxel differed between the cerebral hemispheres (ipsilesional vs. contralesional) with paired sample *t* tests. *T* tests were Bonferroni corrected for multiple comparisons, with α = .012.

To evaluate whether cerebral metabolite concentrations differ between participants with chronic stroke and right‐handed healthy controls, we tested a subset of stroke participants with right hemisphere lesions to control for possible effects of hand dominance on cerebral metabolite concentrations. The ipsilesional right hemisphere in the stroke group was compared to the nondominant right hemisphere in the healthy control group, and conversely the contralesional left hemisphere was compared to the dominant left hemisphere. We performed linear mixed‐effect models to assess the extent to which Group (stroke vs. healthy control) and Hemisphere (ipsilesional/nondominant vs. contralesional/dominant) explained variance in cerebral metabolite concentrations, with age as a covariate and random intercepts fit for each participant to account for repeated measures. Analyses were conducted using the “lmer” and “lmerTest” packages in R (v 4.0.2). Statistical significance of coefficients in the fitted models were assessed with the Satterthwaite approximation. Models were constructed separately for each cerebral metabolite and Bonferroni corrected for multiple comparisons, with α = .010.

To evaluate the impact of stroke on cortical metabolite concentrations, we tested whether adjusted MRS metabolite concentrations (NAA, mI, creatine, choline, GLX) differed between cerebral hemispheres (ipsilesional vs. contralesional) with paired sample *t* tests. *T* tests were Bonferroni corrected for multiple comparisons, with α = .010.

We performed a multiple linear regression analysis to test the degree to which cerebral metabolite concentrations relate to structural damage of the CST, assessed by weighted CST lesion load. Ipsilesional metabolite concentrations were entered as dependent variables with the following independent variables: age, time since stroke (months), and ipsilesional CST lesion load. *R* ^2^ change was calculated for significant predictors. Models were constructed separately for each cerebral metabolite and Bonferroni corrected for multiple comparisons, with α = .010.

We next evaluated whether cerebral metabolite concentrations relate to degree of upper‐extremity motor impairment, assessed by FM scores. FM scores were not normally distributed and showed a bimodal distribution with two clusters of scores at severe and mild/moderate (mild/mod) levels of upper‐extremity impairment (Figure S1), we therefore categorized participants as severe or mild/mod upper‐extremity impairment based on an established FM cut‐off (severe FM ≤ 30; mild/mod: FM > 30; Dobkin & Carmichael, 2016). We next performed logistic regression to explore significant predictors of severe versus mild/mod upper‐extremity impairment. We included impairment status as the dependent variable with the following predictor variables: age, time since stroke, ipsilesional CST lesion load, and ipsilesional and contralesional metabolite concentrations. To reduce the number of predictors in the model, we included only the cerebral metabolites that showed a significant difference between hemispheres across the entire stroke sample (between‐hemisphere *t* test comparisons). Finally, we tested the sensitivity of this model to missing data from stroke participants with large cortical lesions, by multiple imputation of missing data with the MICE package (van Buuren & Groothuis‐Oudshoorn, 2011). Age, time‐since stroke, ipsilesional CST lesion load, and contralesional metabolite concentration were used as predictors for imputation of missing values for ipsilesional metabolite data, using the default predictive mean matching (pmm) method in the MICE package. Multiple imputation was performed for missing ipsilesional data with five iterations and logistic regression models were re‐run. The parameter estimates for the multiply imputed data were pooled, and the significance of pooled estimates with multiply imputed data was computed and compared to logistic regression estimates obtained from observed data.

## 3 RESULTS

### 3.1 Participant characteristics

**FIGURE 2:**
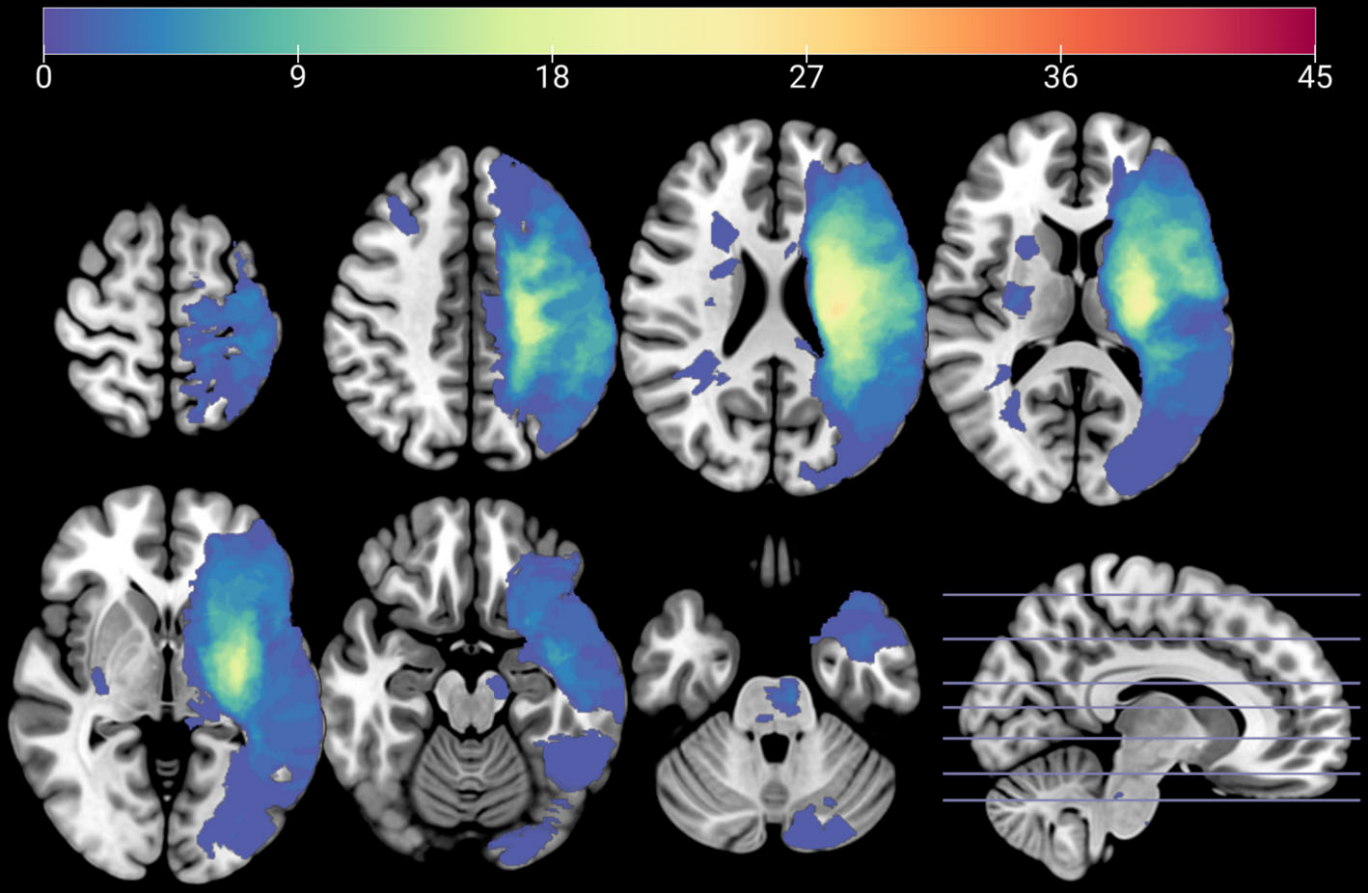
Lesion overlap for 45 individuals with chronic stroke, overlaid onto the MNI T1 template brain in MNI152 space. Symptomatic stroke lesions were flipped to the left hemisphere for visualization. Color scale indicates the number of participants with a stroke involving this voxel

Our sample was comprised of 45 individuals with chronic stroke (means ± SD: Age: 64 ± 11; months since stroke: 60 ± 54; FM score: 39 ± 22; 14 women, 31 men) and 15 healthy older adults (means ± SD: Age: 60 ± 7; 10 women, five men; Table 1). Seven individuals in the stroke group and 11 participants in the healthy control group were included in our previously published MRS study (Jones et al., 2016). Stroke lesions are visualized in Figure 2, and individual demographic data are presented in Table S1. Ipsilesional MRS data was missing from five stroke participants due to extensive cortical lesion involvement (Figure S2). MRS metabolite concentrations did not differ by sex (Table S2).

**TABLE 1:** Participant demographics

	Stroke (*n* = 45)	Stroke subgroups		Healthy control (*n* = 15)
		Severe (*n* = 19)	Mild/mod (*n* = 26)	
Age (years)	64 (11)	61 (12)	67 (9)	60 (7)
Women, *n* (%)	14 (31%)	8 (42%)	6 (23%)	10 (66%)
Time since stroke (months)	60 (54)	57 (48)	63 (58)	
FM score	39 (22)	16 (7)	57 (7)	
Right hemisphere lesion, *n* (%)	23 (51%)	8 (42%)	15 (58%)	
Lesion volume (cc)	34.1 (48.7)	54.3 (56.0)	19.3 (37.1)	
CST lesion load (cc)	3.4 (3.2)	5.3 (3.1)	2.1 (2.6)	

*Note*: Unless otherwise indicated, numbers represent mean and *SD* of the data. The Fugl–Meyer (FM) score is the upper‐extremity portion of the test (out of 66 possible points). Severe and mild/moderate (mild/mod) subgroups were defined by FM score (severe FM ≤ 30; mild/mod: FM > 30; Dobkin & Carmichael, 2016).

Abbreviation: CST, corticospinal tract.

For stroke participants, CSF volume was significantly higher in the ipsilesional relative to the contralesional MRS voxel (*t*_[39]_ = 3.860, *p* < .001) with no significant hemispheric differences in white matter (*t*_[39]_ = −2.557, *p* = .015), gray matter (*t*_[39]_ = 0.416, *p* = .680) or dark non‐CSF tissue volumes (*t*_[39]_ = −1.617, *p* = .114). This hemispheric difference in CSF volume in the MRS voxel was controlled for by adjusting the MRS metabolite signal based on the different T1s and T2s of the tissue and CSF components in the MRS voxel (Macmillan et al., 2016; Meyers et al., 2016).

### 3.2 Metabolite concentrations in individuals with chronic stroke relative to healthy controls

Stroke participants with right hemisphere lesions (*n* = 23) were compared to right‐hand dominant healthy controls (*n* = 15), thus comparisons were made between the ipsilesional/nondominant right hemisphere and the contralesional/dominant left hemisphere. The results of linear mixed‐effect models are presented in Table 2, and mean metabolite concentrations are presented in Table S3. NAA concentrations varied by Group, with lower NAA concentrations in the stroke group relative to healthy controls (*β* = −0.174, *p* = .006), and no significant main effects of Hemisphere or Hemisphere x Group interactions (all *p* > .010). For choline concentrations there was a significant Hemisphere*Group interaction (*β* = −0.124, *p* = .002). Post hoc contrasts revealed that within the stroke group there was lower choline in the ipsilesional hemisphere relative to the contralesional hemisphere (*p* = .008), there were no hemispheric differences in the healthy controls, nor significant differences between the stroke and healthy control groups for either hemisphere (all *p* > .010). All effects of Group, Hemisphere, and Group*Hemisphere interactions were nonsignificant for GLX, creatine, and mI concentrations (all *p* > .010).

**TABLE 2:** Linear mixed‐effect model estimates for the influence of Group (stroke vs. healthy control), Hemisphere (ipsilesional/nondominant vs. contralesional/dominant), and Group*Hemisphere interactions on cerebral metabolite concentrations

	β_(standardized)_	*t* value	*p* value
NAA			
Hemisphere	0.090	1.120	.297
Group	**−0.174**	**8.472**	.006*
Hem*Group	−0.394	3.829	.058
GLX			
Hemisphere	−0.225	0.584	.450
Group	−0.086	0.015	.905
Hem*Group	0.117	0.072	.791
Choline			
Hemisphere	0.039	1.486	.231
Group	0.044	0.224	.639
Hem*Group	**−0.124**	**11.021**	.002*
Creatine			
Hemisphere	0.056	0.064	.803
Group	−0.086	0.781	.383
Hem*Group	−0.073	0.217	.644
mI			
Hemisphere	0.241	0.357	.554
Group	−0.085	1.980	.168
Hem*Group	−0.373	4.194	.048

*Note*: Models were constructed controlling for Age. The reference category for Group was the stroke group, the reference category for Hemisphere was the ipsilesional hemisphere. Note that these models were constructed with a subgroup of stroke participants with right hemisphere lesions (*n* = 23) to control for potential effects of hand dominance on cerebrla metabolite concentrations.

Values in bold are significant at *p <.01.

Abbreviations: GLX, glutamate + glutamine; NAA, N‐acetyleaspartate; mI, myo‐inositol.

### 3.3 Hemispheric differences in MRS metabolites within individuals with chronic stroke

**FIGURE 3:**
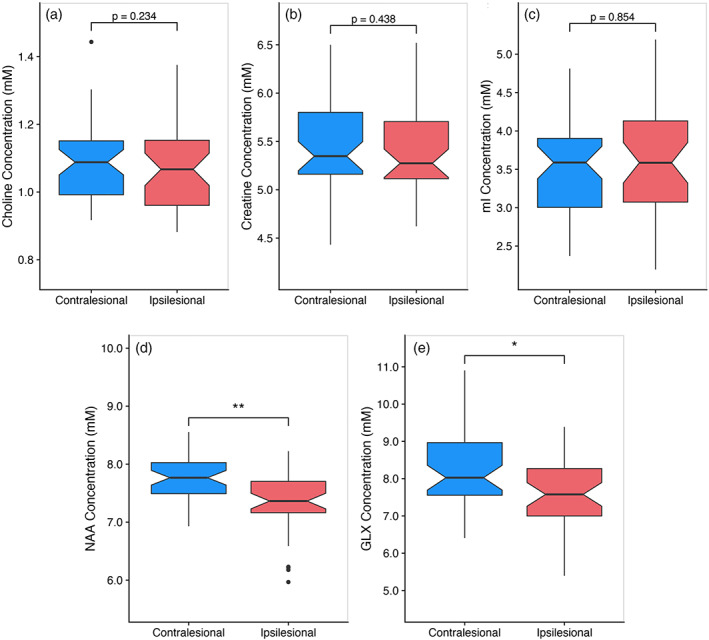
Boxplots illustrating hemispheric differences in cerebral metabolite concentrations after adjustment for tissue composition in the MRS voxel. Hemispheric differences were assessed with paired sample*t* tests. Notched line within the boxplot represents median metabolite concentrations. Boxes and lines represent the interquartile range of the data, with outliers represented by solid black circles. (a) Choline concentration; (b) Creatine concentration; (c) myo‐inositol (mI) concentration; (d) *N*‐acetylaspartate (NAA) concentration; (e) Glutamate + glutamine (GLX) concentration. *p < .01; **p < .001

Results of paired sample *t* test for cortical metabolite concentrations are visualized in Figure 3. Within the stroke group, there was a significant hemispheric difference in concentrations of NAA (*t*_[39]_ = −5.248, *p* < .001) and GLX (*t*_[39]_ = −3.115, *p* = .003), with significantly lower metabolite concentrations in the ipsilesional hemisphere relative to the contralesional hemisphere. There were no significant differences between hemispheres for choline, creatine, or mI concentrations (all *p* > .010). NAA and GLX concentrations were used for subsequent logistic regression analyses with motor impairment.

### 3.4 Relationships between cerebral metabolites and CST damage

The results of linear regression models relating ipsilesional cerebral metabolite concentrations to CST lesion load are presented in Table 3. After controlling for age and time since stroke, ipsilesional CST lesion load explained a significant amount of variance in ipsilesional NAA concentration (*β* = −0.305, *p* = .006, Δ*R* ^2^ = 0.189, *F*_[1,36]_ = 8.607, *p* = .006). Ipsilesional CST lesion load did not explain a significant amount of variance in ipsilesional GLX, choline, creatine, or mI concentrations (all *p* > .010).

**TABLE 3:** Linear regression models evaluating relationships between ipsilesional corticospinal tract lesion load and ipsilesional cerebral metabolite concentrations in individuals with chronic stroke

DV	Predictors	*R* ^2^	*F*_(3,36)_	*p* value	*CST_LL*	
					β_(standardized)_	*p* value
NAA	Age + TSS + CST_LL	0.210	3.183	0.035	**−0.305**	**.006** *
GLX	Age + TSS + CST_LL	0.111	1.419	0.233	0.243	.150
Choline	Age + TSS + CST_LL	0.114	1.549	0.218	−0.011	.670
Creatine	Age + TSS + CST_LL	0.042	0.523	0.67	−0.010	.912
mI	Age + TSS + CST_LL	0.127	1.744	0.176	0.221	.117

*Note*: Models were constructed controlling for age and time since stroke (TSS; months).

Values in bold are significant at *p <.01.

Abbreviations: CST_LL, Corticospinal tract lesion load; DV, dependent variable; GLX, glutamate + glutamine; mI, myo‐inositol; NAA, *N*‐Acetylaspartate.

### 3.5 Relationships between cerebral metabolites and upper‐extremity impairment

The results of logistic regression models between motor impairment (severe and mild/mod) and cerebral metabolites are presented in Table 4. The first logistic regression model included all observed data in our dataset. After controlling for age and time since stroke, NAA and GLX concentrations did not relate to upper‐extremity impairment (FM score) for either hemisphere (all *p* > .010). Ipsilesional CST lesion load was a significant predictor of upper‐extremity impairment (*β* = 1.723, *p* = .026), with higher CST lesion load in severe individuals relative to mild/mod individuals. Next, we evaluated whether observed relationships between cortical metabolite and upper extremity impairment were impacted by missing data from participants with large cortical lesions (*n* = 5). Individuals with missing MRS data due to large cortical involvement of the lesion (*n* = 5) had more severe motor impairment (*t*_[43]_ = 2.506, *p* = .016) and greater CST lesion load (*t*_[43]_ = −4.361, *p* < .001) relative to the complete sample. We re‐ran logistic regression models as a sensitivity analysis to evaluate whether participants with missing data due to large cortical lesions (*n* = 5) significantly impacted parameter estimates of ipsilesional MRS metabolites for predicting motor impairment severity. After multiple imputation of missing cortical metabolite data parameter estimates from each of the five imputed datasets were pooled (see Figure S3 and Table S4 for descriptors of imputed datasets). Final model estimates revealed that after multiple imputation ipsilesional NAA and GLX remained nonsignificant predictors of severe impairment (all *p* > .010), and ipsilesional CST lesion load remained a significant predictor of severe impairment (*β* = 1.532, *p* = .026).

**TABLE 4:** Logistic regression for severe versus mild/moderate upper extremity impairment, evaluating candidate predictors of upper extremity impairment outcomes

Predictor	Observed data			Imputed data		
	β_(standardized)_	*z* value	*p* value	β_(standardized)_	*z* value	*p* value
Age	−0.082	−0.149	.882	−0.549	−1.036	.309
TSS	−1.061	−1.795	.073	−1.070	−1.965	.057
Ipsilesional NAA	−0.705	−1.217	.224	−0.598	−0.997	.329
Contralesional NAA	0.977	1.582	.114	0.905	1.683	.102
Ipsilesional GLX	0.957	1.707	.088	0.636	1.146	.262
Contralesional GLX	0.963	1.829	.067	1.051	2.012	.052
CST_LL	**1.723**	**2.229**	.026*	**1.532**	**2.330**	.026*

*Note*: For our observed data (*n* = 40), CST lesion load was the only significant predictor of upper extremity impairment and cortical metabolites did not relate to upper extremity impairment. This result was robust to the imputation of missing MRS data (*n* = 5) from individuals with large cortical lesions. Values in bold are significant at *p* <.01; * *p* <.05.

## 4 DISCUSSION

Understanding the neurobiological processes of stroke recovery is critical if we are to improve patient outcomes. In this study, we examined cerebral metabolite concentrations in vivo from the primary sensorimotor cortex of individuals with chronic stroke. We found that NAA concentrations in the sensorimotor cortex are impacted by stroke; demonstrated by reduced concentration bilaterally compared to healthy controls, reduced concentration in the ipsilesional relative to contralesional hemisphere within the stroke sample, and a negative relationship between CST lesion load and ipsilesional NAA concentration. No other cerebral metabolite showed this consistent pattern of effects across our analyses. Despite the clear stroke‐related effects on NAA, there was no relationship between cortical NAA and severity of motor impairment in our sample. This finding was robust to any effect of missing data from individuals with large cortical lesions.

NAA is one of the most abundant metabolites in the brain, and is localized to neurons and their associated oligodendrocytes (Nordengen, Heuser, Rinholm, Matalon, & Gundersen, 2015). Several lines of evidence indicate that NAA is a sensitive marker of stroke‐related damage: NAA concentration decreases in the acute stage after reperfusion therapy in penumbral tissue with poor reperfusion (Bivard et al., 2014), and peri‐infarct NAA shows a progressive decline from the acute to subacute period poststroke (Maniega et al., 2008; Mazibuko et al., 2020). In the chronic phase of recovery, our data validate previous findings of lower ipsilesional NAA in spared cortex remote to the lesion site (Carlson et al., 2017; Cirstea et al., 2011; Craciunas et al., 2013; Jones et al., 2016). Additionally, we report a novel finding that lower ipsilesional NAA concentrations in primary sensorimotor cortex relate to higher lesion load in the ipsilesional CST. This suggests a loss of cortical neuronal density or metabolism that is specific to the location of stroke injury. Low ipsilesional NAA concentrations may be caused by Wallerian degeneration (Egorova et al., 2020), or long‐term metabolic depression of cortical tissue with connectivity to the stroke lesion (Wang et al., 2019). However, the function of NAA in the central nervous system remains controversial and changes in NAA concentrations have been interpreted as reflecting the number or integrity of neurons in the target tissue, or their metabolic rate (Moffett, Ross, Arun, Madhavarao, & Namboodiri, 2007). Future investigations into the basic biochemistry of NAA in the central nervous system might help to reveal mechanisms of long‐term cortical adaptation to stroke injury. These investigations are a necessary complement to future in vivo studies of NAA with MRS in humans poststroke. Based on our findings, and results from these previous studies, NAA concentrations demonstrate sensitivity to stroke‐related damage. NAA may be an informative mechanistic marker for future investigations in both basic and clinical research.

Contrary to our hypothesis, we did not observe a relationship between ipsi‐ or contralesional NAA concentrations and chronic motor impairment (severe vs. mild/moderate impairment indexed by FM score). Our study included the largest sample size to date testing relationships between cortical metabolites and motor impairment, and we did not observe any relationship between the two variables including after imputation of missing data from individuals who presumably should show the strongest relationship between cortical metabolite levels and motor impairment (due to profound cortical lesion involvement). Previous studies have found that cortical NAA concentration relates to motor outcomes in individuals with chronic stroke (Cirstea et al., 2011, 2014; Craciunas et al., 2013; Jones et al., 2016). Compared to previous research on this topic, our study included a more diverse range of stroke lesion types, and a more stringent correction for cortical atrophy in MRS metabolite estimates; any of these factors may explain the lack of a significant relationship with motor impairment in the present report. We also included a larger MRS voxel relative to some previous reports (Cirstea et al., 2011, 2014; Craciunas et al., 2013), but similar to others (Carlson et al., 2017; Jones et al., 2016). Larger voxel size increases signal to noise ratio therefore improving our changes to detect a physiologically relevant metabolite signal, but we lose some degree of anatomical specificity as a result. Tissue from the premotor cortex was likely captured in our voxel, however previous MRS studies employing voxels specific to premotor cortices have also reported relationships between ipsilesional premotor NAA and chronic upper‐extremity impairment (Cirstea et al., 2012; Craciunas et al., 2013). Unlike ipsilesional metabolite concentrations, weighted ipsilesional CST lesion load did significantly relate to upper extremity impairment which is consistent with multiple previous reports (Feng et al., 2015; Findlater et al., 2019; Zhu, Lindenberg, Alexander, & Schlaug, 2010). The CST receives projections from multiple cortical regions, including primary motor, somatosensory, and premotor regions (Schulz et al., 2012), thus descending CST integrity is perhaps more likely to relate to motor impairment than metabolite concentrations within the hand knob region. We conclude that NAA concentration appears to be sensitive to the effects of stroke and may provide useful mechanistic information about cortical response to CST injury. However, NAA concentration is currently not a robust candidate for translation to clinical practice as a predictor of upper‐extremity impairment.

GLX concentrations were also lower in the ipsilesional, relative to contralesional, sensorimotor cortex. GLX is the combined spectral peaks of both glutamate and glutamine; glutamate is the most abundant excitatory neurotransmitter in the central nervous system and is converted to glutamine for storage in glial cells (Ramadan et al., 2013). Low ipsilesional GLX in individuals with chronic stroke provides neurochemical evidence for a long‐held model of imbalance in cortical excitation between the cerebral hemispheres in individuals with chronic stroke (Edwards et al., 2013; Murase, Duque, Mazzocchio, & Cohen, 2004; Volz et al., 2015; Ward, Brown, Thompson, & Frackowiak, 2003). According to this model, we would also expect that either ipsi‐ or contralesional glutamate concentrations would relate to motor impairment (i.e., greater contralesional excitation or less ipsilesional excitation should relate to more severe motor impairment). However, this was not reflected in our data. We observed no group, and importantly, no hemisphere by group interaction when we included the healthy control participants and the stroke group for the GLX metabolite concentrations. Further neither ipsilesional nor contralesional GLX related to chronic motor impairment. Cirstea et al., 2011, reported a negative relationship between ipsilesional GLX and severity of motor impairment (i.e., more severe impairment was associated with higher ipsilesional GLX), which is also incongruent with predictions derived from the interhemispheric competition model (Murase et al., 2004). It may be that inhibitory, rather than excitatory, signaling is more critical for the interhemispheric inhibition model, or as others have suggested, this model may have to be revised based on individualized factors such as time since stroke, and impairment severity (McCambridge, Stinear, & Byblow, 2018; Neva, Hayward, & Boyd, 2019; Xu et al., 2019).

We did not observe differences in mI levels, either between hemispheres within the stroke group or between the stroke participants and healthy controls. This is contrary to previous findings of elevated mI in the sensorimotor cortex of individuals with chronic stroke (Cirstea et al., 2011; Craciunas et al., 2013). MI is principally located in glial cells (Brand, Richter‐Landsberg, & Leibfritz, 1993), and elevated mI levels have previously been interpreted for evidence of gliosis (Glanville, Byers, Cook, Spence, & Palmer, 1989) or for glial involvement in poststroke plasticity (Cirstea et al., 2011). Our data did not support these previous findings and therefore the question of detection of glial involvement by MRS in chronic stroke should be re‐evaluated in future MRS studies.

Controlling for tissue composition is essential when analyzing and interpreting MRS data, especially where cortical atrophy is present such as in typical aging and after stroke. In this study, trained researchers manually placed the MRS voxels aiming for symmetrical placement between the cerebral hemispheres. However, we still observed significantly more CSF occupying the MRS voxel in the ipsilesional relative to the contralesional voxel. Cortical tissue loss after stroke is expected to be asymmetric because of retrograde degeneration of regions connected to the stroke infarct (Cheng et al., 2015). Changes in the functional or metabolic characteristics of ipsilesional tissue must therefore be considered cautiously with respect to the loss of total neuronal matter within the ipsilesional hemisphere, especially when testing for hemispheric asymmetries. Some previous MRS studies have corrected for this issue by dividing metabolite concentrations by a proportion of the voxel occupied by brain tissue or CSF (Cirstea et al., 2011, 2014; Craciunas et al., 2013); yet this approach does not take into account the variance of metabolite concentrations present in different brain tissues types (i.e., gray matter, white matter, and lesion). Another common approach for adjusting MRS metabolite concentrations is to normalize metabolites of interest as a ratio against metabolites that are assumed to be stable across the brain, typically creatine and choline (Jansen et al., 2006). Relative metabolite concentrations are simpler and less time consuming to implement than adjusting absolute metabolite concentrations. However, with this approach observed changes in metabolite ratios may result from shifts in the metabolite of interest, the “control” metabolite, or both. Our approach instead corrected for differences in water T1 and T2 relaxation of brain tissue types (Macmillan et al., 2016; Meyers et al., 2016) in order to perform a stringent adjustment of MRS concentrations for tissue composition in the MRS voxel. Therefore, our findings are unlikely to be an artefactual difference resulting from expected loss of cortical tissue. Future MRS studies should analyze absolute metabolite concentrations with adjustment for voxel tissue composition. This is especially important in cerebrovascular or neurodegenerative disease populations with anticipated cortical atrophy.

Participants with large cortical lesions present a challenge for any study measuring cortical variables. Importantly, individuals with large cortical lesions in our sample had significantly greater motor impairment and greater extent of damage to the CST making their inclusion critical for the identification of potential relationships between brain biomarkers and upper‐extremity motor outcomes (Hayward et al., 2017). We addressed this issue with a multiple imputation method to replace missing ipsilesional values and model pooled parameter estimates for imputed data. With this analysis, we were able to confirm that the observed relationships between brain markers and motor impairment were robust to the presence of missing data from individuals with large cortical lesions. Missing data from more severely impaired individuals with large lesions are common in stroke research, however their data are missing not‐at‐random making them important to consider when validating brain‐behavior relationships. We suggest multiple imputation as a useful approach to develop theoretical models of stroke recovery through inclusion of individuals with missing data due to extensive ipsilesional damage.

Strengths of our study include the large sample size relative to previous MRS papers, the stringent adjustment of MRS metabolites estimates for proportion of tissue in the MRS voxel, and the sensitivity analysis to examine the impact of missing data on MRS metabolite and motor impairment relationships. Our study also has several limitations. The MRS voxel was placed over the anatomical representation of the hand knob, rather than fMRI guided MRS voxel placement over regions with hand related activity. This, as well as variations in the size of the MRS voxel between studies, may contribute to discrepancies between our findings and those of previous MRS studies (Carlson et al., 2017; Cirstea et al., 2011, 2014; Craciunas et al., 2013). We had a small sample of healthy control data, as this was a secondary aim of the paper. A small portion of our participants (*n* = 6) had small infarcts in the contralesional hemisphere, which may be covert lacunar strokes or bilateral infarcts. This speaks to the representative nature of our stroke sample; however, this may have reduced our power for between hemisphere contrasts. We did not collect data on whether the ipsilesional hemisphere for the stroke participants occurred in the dominant or nondominant hemisphere pre‐stroke, which may contribute to variability in the data. Other factors such as depression and cardiometabolic comorbidities could also impact cortical metabolites after stroke, as this was beyond the scope of the present study this should be investigated in future research. Finally, this study is cross‐sectional in design, and our findings will need to be validated over the course of stroke recovery in a longitudinal study.

In summary, our data suggests that cortical NAA concentrations are low in chronic stroke; both relative to concentrations observed in healthy controls and in the ipsilesional hemisphere relative to the contralesional hemisphere within individuals with chronic stroke. Furthermore, ipsilesional NAA concentration relates negatively to extent of injury to the CST, with decreasing cortical NAA with increasing extent of injury to the CST. We adjusted our MRS data for tissue composition in the MRS voxel, and therefore our results represent stroke‐related effects on NAA concentrations that are independent of any concurrent loss of cortical gray or white matter volume. Future MRS research may lend informative mechanistic insights into the neurobiological processes of stroke recovery. However, given the current technical demands of MRS, and the weak associations between cortical neurochemical concentrations and motor impairment, we conclude that MRS currently has limited evidence for translation to the clinic as a biomarker of chronic poststroke motor outcomes.

## CONFLICT OF INTEREST

The authors declare no conflicts of interest.

## Supporting information


**DATA S1**: Supporting information

## Data Availability

The data that support the findings of this study are available from the corresponding author upon reasonable request.
