# Supplementary material for: Cortical N‐acetylaspartate concentrations are impacted in chronic stroke but do not relate to motor impairment: A magnetic resonance spectroscopy study
Source: Hum Brain Mapp. 2021 May 3;42(10):3119–30. doi: 10.1002/hbm.25421 (PMC8193507; doi:10.1002/hbm.25421)
Supplement: Supplementary file 1 — DATA S1: Supporting information [file HBM-42-3119-s001.docx]

**Supplementary Materials**

**MRS Metabolites Adjustment**

Metabolite concentrations from LCModel were adjusted for tissue composition within the MRS voxel as follows:

$Adjusted[MET]= \frac{{[MET]}_{LCModel}\times WCONC \times ATTH20}{(1-F_{CSF)}}$

$$WCONC=55556mM \times[{WC}_{WM}\times F_{WM}+ {WC}_{GM}\times F_{GM}+ {WC}_{CSF}\times F_{CSF}]$$

$ATTH2O= F_{WM} \times\left[ e^{-\frac{TE}{{T2}_{WM}}}\left( 1-e^{-\frac{TR}{{T1}_{WM}}} \right) \right]+F_{GM}\times\left[ e^{-\frac{TE}{{T2}_{GM}}}\left( 1-e^{-\frac{TR}{{T1}_{GM}}} \right) \right]+F_{CSF}\times\left[ e^{-\frac{TE}{{T2}_{CSF}}}\left( 1-e^{-\frac{TR}{{T1}_{CSF}}} \right) \right]$

Where:

WCtype is the percent concentration wet weight of water in the tissue type

Ftype is the volume fraction of each tissue type

TE is the echo time in seconds

T2type is the T2 relaxation time in seconds of water protons in each tissue type TR is the repetition time in seconds

TR is the repetition time in seconds

T1type is the T1 relaxation time in seconds of water protons in each tissue type

The water content, T_1_ and T_2_ relaxation values used to determine the voxel water concentration of the unsuppressed water peak, using literature values (Meyers et al, *J. Magn. Reson. Imaging. 2016;44:296–304*; Macmillan et al, *Mult. Scler.* 2016;22:112–116) as follows:

|  | WM | Lesion | GM | CSF |
| --- | --- | --- | --- | --- |
| Water concentration | 72% | 82.7% | 82% | 99% |
| T_1_ | 1060 | 1230 | 1470 | 3000 |
| T_2_ | 74 | 93 | 110 | 500 |

**
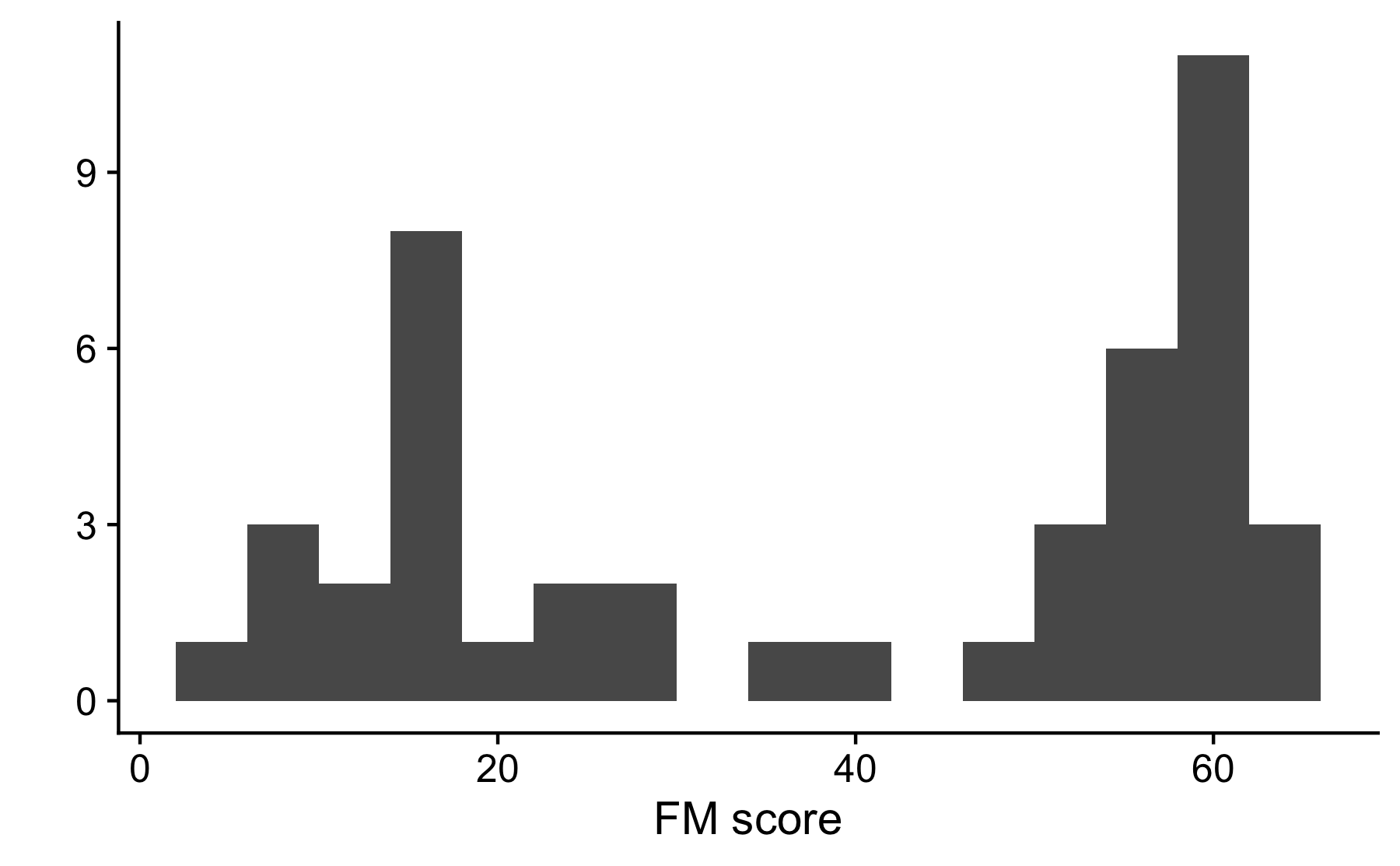
**

Supplementary Figure 1: Histogram of Fugl-Meyer (FM) score, measuring upper extremity impairment in a cohort of 45 individuals with chronic stroke. FM scores showed a non-normal and bimodal distribution with 2 groups clustering at severe (≤30 FM) and mild/moderate (>30 FM) levels of impairment.

Supplementary Figure 2: T1-weighted images with contralesional voxels for 5 participants with missing ipsilesional data. For these individuals it was not possible to quantify ipsilesional MRS data due to large cortical involvement stroke lesions. Numbers indicate participant ID (see Supplementary table 1).

| ID | Sex | Age | TSS | FM | Lesioned Hemisphere | Missing Ipsilesional data |
| --- | --- | --- | --- | --- | --- | --- |
| 1 | M | 70 | 14 | 4 | R |  |
| 2 | M | 57 | 94 | 7 | R |  |
| 3 | M | 62 | 85 | 8 | L | Y |
| 4 | F | 73 | 35 | 8 | L | Y |
| 5 | F | 69 | 19 | 11 | R |  |
| 6 | F | 36 | 89 | 11 | R | Y |
| 7 | M | 58 | 25 | 15 | L |  |
| 8 | F | 65 | 21 | 15 | L |  |
| 9 | F | 67 | 119 | 16 | R |  |
| 10 | M | 61 | 91 | 16 | L |  |
| 11 | M | 51 | 22 | 16 | L |  |
| 12 | M | 57 | 23 | 16 | R | Y |
| 13 | M | 81 | 96 | 16 | L |  |
| 14 | M | 33 | 33 | 18 | L |  |
| 15 | M | 63 | 41 | 22 | R |  |
| 16 | F | 58 | 18 | 25 | L |  |
| 17 | M | 55 | 20 | 25 | R |  |
| 18 | F | 78 | 43 | 27 | L |  |
| 19 | F | 60 | 196 | 30 | L |  |
| 20 | F | 56 | 27 | 35 | R |  |
| 21 | F | 54 | 77 | 41 | R |  |
| 22 | M | 76 | 152 | 48 | R | Y |
| 23 | M | 59 | 270 | 52 | L |  |
| 24 | M | 70 | 18 | 54 | L |  |
| 25 | M | 60 | 23 | 54 | R |  |
| 26 | M | 68 | 112 | 56 | R |  |
| 27 | F | 71 | 83 | 56 | L |  |
| 28 | M | 64 | 94 | 56 | R |  |
| 29 | M | 69 | 15 | 57 | R |  |
| 30 | F | 66 | 27 | 58 | R |  |
| 31 | M | 71 | 20 | 58 | R |  |
| 32 | M | 76 | 72 | 59 | L |  |
| 33 | M | 82 | 12 | 59 | L |  |
| 34 | M | 79 | 18 | 59 | L |  |
| 35 | M | 73 | 142 | 60 | L |  |
| 36 | M | 85 | 35 | 60 | R |  |
| 37 | M | 59 | 54 | 61 | L |  |
| 38 | M | 67 | 82 | 62 | R |  |
| 39 | M | 76 | 81 | 62 | R |  |
| 40 | M | 65 | 67 | 62 | R |  |
| 41 | M | 55 | 24 | 62 | L |  |
| 42 | M | 62 | 20 | 62 | L |  |
| 43 | F | 50 | 37 | 63 | L |  |
| 44 | F | 68 | 52 | 65 | R |  |

| Metabolite | Hemisphere | Male mean (SD) | Female mean (SD) | t_(53)_ | p |
| --- | --- | --- | --- | --- | --- |
| NAA | Contralesional | 7.40 (0.53) | 7.58 (0.58) | 1.206 | 0.233 |
|  | Ipsilesional | 7.06 (0.73) | 7.34 (0.61) | 1.422 | 0.161 |
| Choline | Contralesional | 1.05 (0.11) | 1.02 (0.15) | -1.191 | 0.238 |
|  | Ipsilesional | 1.04 (0.13) | 1.02 (0.13) | -0.973 | 0.335 |
| Creatine | Contralesional | 5.29 (0.48) | 5.23 (0.53) | -0.684 | 0.497 |
|  | Ipsilesional | 5.27 (0.54) | 5.15 (0.49) | -1.434 | 0.158 |
| mI | Contralesional | 3.53 (0.58) | 3.35 (0.68) | -1.242 | 0.219 |
|  | Ipsilesional | 3.56 (0.78) | 3.43 (0.62) | -0.840 | 0.405 |
| GLX | Contralesional | 8.00 (1.00) | 7.78 (1.11) | -0.890 | 0.377 |
|  | Ipsilesional | 7.41 (0.93) | 7.31 (0.84) | -0.834 | 0.408 |

**Supplementary Table 1:** Participant demographic information. TSS: time since stroke (months); FM: Fugl-Meyer score; R: Right Hemisphere; L: Left Hemisphere;

**Supplementary Table 2:** Sex differences in adjusted cerebral metabolite concentrations across all observed data. NAA: N-Acetylaspartate; mI: myo-Inositol; GLX: Glutamate + Glutamine

| Metabolite | Hemisphere | RHem stroke mean (SD) | Healthy Control mean (SD) |
| --- | --- | --- | --- |
| NAA | Contralesional (Dominant) | 7.62 (0.34) | 7.76 (0.64) |
|  | Ipsilesional (Non-Dominant) | 7.32 (0.52) | 7.85 (0.39) |
| Choline | Contralesional (Dominant) | 1.08 (0.13) | 1.04 (0.14) |
|  | Ipsilesional (Non-Dominant) | 1.01 (0.10) | 1.08 (0.12) |
| Creatine | Contralesional (Dominant) | 5.33 (0.43) | 5.41 (0.50) |
|  | Ipsilesional (Non-Dominant) | 5.32 (0.41) | 5.46 (0.51) |
| mI | Contralesional (Dominant) | 3.52 (0.54) | 3.63 (0.67) |
|  | Ipsilesional (Non-Dominant) | 3.37 (0.56) | 3.88 (0.76) |
| GLX | Contralesional (Dominant) | 7.94 (0.83) | 8.11 (1.34) |
|  | Ipsilesional (Non-Dominant) | 7.81 (0.76) | 7.88 (0.76) |

Supplementary Table 3: Means and standard deviations (SD) of cortical metabolite concentrations in individuals with right hemisphere lesions (RHem; n=23) compared to right-handed healthy adult controls (n=15). The ipsilesional hemisphere in individuals with right-hemisphere lesions was compared to the non-dominant (right) hemisphere in healthy controls. NAA: N-Acetylaspartate; mI: myo-Inositol; GLX: Glutamate + Glutamine


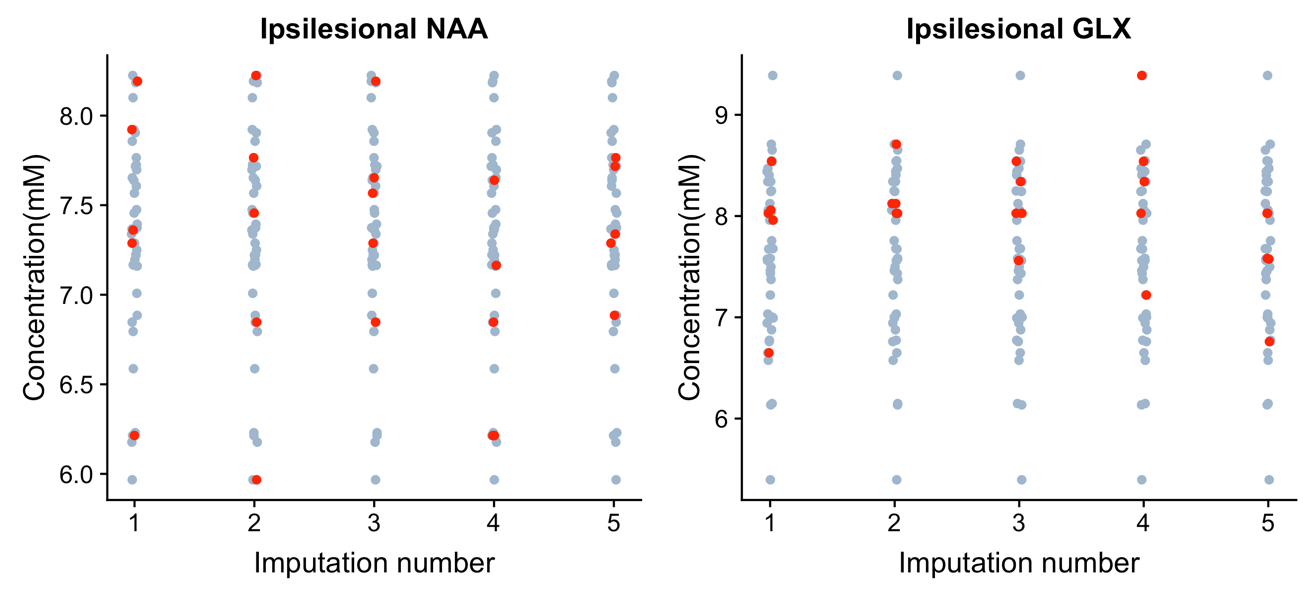


**Supplementary Figure 3:** Spread of imputed data across 5 imputed datasets. The x-axis represents the imputed dataset number. The y-axis is metabolite concentrations for ipsilesional NAA (left) and GLX (right). Blue points are observed data, red points are imputed data.

|  | NAA |  |  | GLX |  |  |
| --- | --- | --- | --- | --- | --- | --- |
|  | mean | SD |  | mean | SD |  |
| Observed data | 7.33 | 0.55 |  | 7.60 | 0.83 |  |
| Imputation 1 | 7.33 | 0.57 |  | 7.63 | 0.81 |  |
| Imputation 2 | 7.32 | 0.59 |  | 7.67 | 0.81 |  |
| Imputation 3 | 7.35 | 0.55 |  | 7.66 | 0.81 |  |
| Imputation 4 | 7.27 | 0.58 |  | 7.68 | 0.85 |  |
| Imputation 5 | 7.33 | 0.53 |  | 7.60 | 0.80 |  |

**Supplementary Table 4:** Means and standard deviations (SD) for observed and imputed data across 5 imputed datasets. Data presented are ipsilesional concentrations of N-Acetylaspartate (NAA) and glutamate+glutamine (GLX)
